# The Ketogenic Diet and Potential Micronutrient Risks in Drug-Resistant Epilepsy Management: A Literature Review

**DOI:** 10.3390/nu18071081

**Published:** 2026-03-27

**Authors:** Bhavini Singh, Paige Botten, Katherine P. Richardson, Chaston Weaver, Sharad Purohit

**Affiliations:** 1 Medical College of Georgia, Augusta University, Augusta, GA 30912, USA; bhsingh@augusta.edu (B.S.); chweaver@augusta.edu (C.W.); 2 Department of Nutrition and Dietetics, College of Allied Health Sciences, Augusta University, Augusta, GA 30912, USA; paige.botten@va.gov; 3 Philadelphia College of Osteopathic Medicine, Georgia Campus, Suwanee, GA 30024, USA; kr3909@pcom.edu; 4 Center for Biotechnology and Genomic Medicine, Augusta University, Augusta, GA 30912, USA; 5 Department of Gynecology and Obstetrics, Medical College of Georgia, Augusta University, Augusta, GA 30912, USA

**Keywords:** neurological disorders, drug-resistant epilepsy, seizures, nutritional deficiencies, clinical diets, ketogenic diet

## Abstract

The ketogenic diet (KD) is a critical, evidence-based intervention within medical nutrition therapy for managing neurological disorders. In this article, we reviewed the published research on the efficacy of the ketogenic diet and its variations in treating epilepsy, particularly for patients unresponsive to anti-epileptic drugs. The literature review was performed on PubMed between 2022 and 2025. The review of clinical studies across various age groups reveals that, while the KD is effective for both focal and generalized seizures, infants often achieve higher rates of seizure freedom compared to adults, potentially due to better dietary compliance. Despite its success, the restrictive nature of the diet presents significant challenges for individuals suffering from epilepsy. The key challenges that reduce compliance over time include side effects, such as gastrointestinal issues, potential for malnutrition, and a high risk of micronutrient deficiencies. The role of the registered dietitian is paramount in this interdisciplinary approach, ensuring personalized education by monitoring growth and adjusting nutritional plans to optimize health outcomes for children unresponsive to anti-epileptic drugs. Ultimately, integrating MNT with traditional pharmacological or surgical treatments offers the most promising path for significant seizure reduction and improved quality of life for epileptic patients.

## 1. Introduction

Clinical diets, often referred to as therapeutic diets, are a common and important component of treatment plans for numerous health conditions [1]. In some conditions, such as Celiac Disease, a carefully managed diet can be the primary and sole treatment for disease management [2,3,4,5]. Implementing a clinical diet involves careful management of nutrition, which entails medical nutrition therapy (MNT). MNT is an evidence-based practice executed by a registered dietitian (RD) who assesses a patient’s overall health to determine the most suitable diet for their condition [6,7].

Evidence-based general nutrition guidelines have been established for several health conditions. Medical nutritional therapy implements the patient-centered care model by optimizing individual patient health, acknowledging cost-effectiveness, and improving overall quality of life when other medications and treatments are ineffective [8].

This review presents a literature search on MNT-based clinical diets for epilepsy from the perspective of an RD. Epilepsy is a neurological disorder characterized by recurrent seizures [9,10]. Seizures associated with epilepsy are classified into two primary categories: focal onset seizures and generalized onset seizures [11]. Focal seizures, also referred to as partial seizures, originate in one hemisphere of the brain, whereas generalized seizures involve both hemispheres [11].

Epilepsy presents with a range of clinical manifestations and is primarily defined by seizure type. The condition may result from genetic or congenital factors and perinatal disorders, such as cerebral palsy or brain injury occurring before or after birth [9,10]. Epilepsy may also develop later in life and can be precipitated by events, including traumatic brain injury, brain tumors, or cerebrovascular disease [10]. Dietary interventions, particularly the ketogenic diet (KD) and its variations such as the modified Atkins diet (MAD) [12,13,14], have demonstrated therapeutic benefits in the management of epilepsy and related conditions.

**Figure 1:**
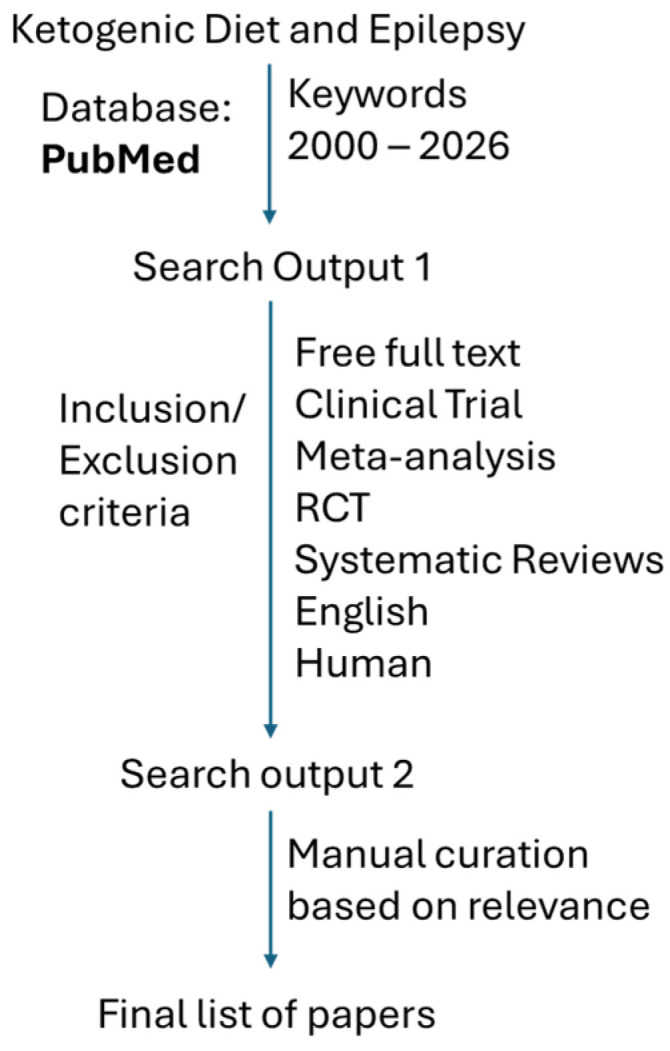
**PRISMA flowchart displaying the databases searched and date ranges used for this literature review**. Keywords used and the number of papers screened and retained in this literature review are presented in Table 1. RCT: randomized clinical trial.

The literature search was performed between 2022 and 2025 on PubMed for the studies published on KD and epilepsy from 2000 onwards. The keywords used for the search are “medical nutrition therapy AND epilepsy”, “Ketogenic diet and epilepsy”, “clinical diets AND epilepsy”, “seizure AND medical nutritional therapy”, and “seizure AND clinical diets”. Additional filters such as “availability of free full text”, “Clinical Trial”, “Meta-Analysis”, “Randomized Controlled Trial”, and “Systematic Review” were applied. Additionally, these research papers published in “English” and “Human” Subjects were filtered. The PRISMA flow chart shows the process of the search performed (Figure 1), and the number of records screened and retained are presented in Table 1. For the final list after all the filters and relevance, we selected papers to review on clinical trials on KD and epilepsy only (see Table 1).

## 2. Epilepsy and the Ketogenic Diet

Fasting was recognized as an effective means to reduce seizures as far back as ancient Greece and early Christian texts [15,16]. In the early 20th century, the observation that fasting induced ketosis led to the development of the classic ketogenic diet—a high-fat, low-carbohydrate diet with adequate-protein intake. The ketogenic diet mimics the metabolic effects of starvation and induces sustained ketosis with anticonvulsant properties [17,18,19]. Over time, health professionals have found many epileptic patients to be unresponsive to medications, requiring other forms of treatment. At the turn of the twentieth century, the scientific community became interested in a high-fat, low-protein, and low-carbohydrate diet in the hunt for new ways to treat seizures, both pharmacologically and holistically. In 1912, the first effective anti-convulsive medication was introduced to medical practice. However, over time, health professionals noted that some patients were unresponsive to the medications, warranting further investigation into diet-based therapies. Rollin Turner Woodyatt’s clinical investigation, in 1921, was one of the first [19]. Followed by Russel Wilder, who, in the same year, studied the association between diet and seizure control [18]. Later, the classic ketogenic diet was proven and established by Mynie Peterman, providing seizure control in 95% of study patients [19]. The ketogenic diet became the sole treatment for epilepsy until anti-convulsant medications were developed in the early 20th century (1912–1938) [20]. After years of successful implementation, the ketogenic diet is an established MNT and plays a vital role in epilepsy management, offering effective, non-pharmacologic options for seizure control.

**Figure 2:**
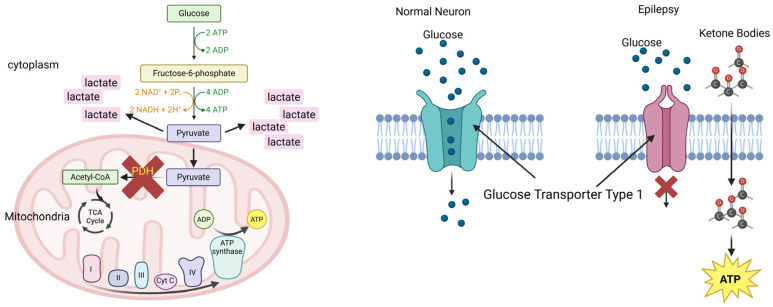
**Metabolic changes in brain cells leading to “electrical storms” in epilepsy**. Defects in the pyruvate dehydrogenase complex break the bridge between glycolysis and the citric acid cycle. The energy generation switches to the less efficient pyruvate-to-lactic acid pathway, causing buildup of lactic acid (**left**). Defects or inactivation of glucose transporter-1 in neurons restrict the entry of glucose required for energy production in neurons (**right**). Both pathways contribute to impaired ATP production and the accumulation of toxic metabolites, to trigger epileptic activity. Created in BioRender. Purohit, S. (2026) https://BioRender.com/c6fzt33 (accessed on 28 January 2026).

While the exact mechanism of how the ketogenic diet reduces seizure episodes is not fully understood, there are many theories [21,22]. The influence of a ketogenic diet on seizure reduction can also vary based on the etiology of epilepsy. In this review, we will focus on the metabolic bypass mechanism and its relationship with the KD diet. For example, epilepsy can result from pyruvate dehydrogenase deficiency [23], a metabolic disorder in which glycolysis cannot occur at a rate needed to meet the energy demands of the brain, causing a buildup of pyruvate and lactate [24]. Here, the ketogenic diet is effective as it bypasses carbohydrate metabolism and glycolysis; instead, ketone production from proteins and fat is used as an energy source. Glucose transporter type 1 deficiency syndrome is another source of seizures, as glucose cannot cross the blood-brain barrier [25]. In this condition, the ketogenic diet is beneficial as ketone bodies can cross the blood-brain barrier without a transporter and can be used for metabolic activity by the brain [25]. At present, the ketogenic diet has been consistently reported to be beneficial, with more than 70% of patients showing positive responses [20,21,25]. Furthermore, by introducing a long-chain triglyceride diet, adequate levels of polyunsaturated fatty acids are made available for the developing brain [26] (Figure 2).

The ketogenic diet has been deemed to be an effective treatment for multiple epileptic conditions, especially for patients who are resistant to anti-epileptic drugs (AEDs). The ketogenic diet has evolved into four different variations, improving its adaptability and potential effectiveness as a treatment for various patients. The variations include the classic ketogenic diet, modified Atkins diet, medium-chain triglycerides ketogenic diet, and low glycemic index treatment [27]. The variations differ in several ways, including the fat-to-carbohydrate and protein ratio, the type of fat and carbohydrates provided, among others. Evidence indicates that the ketogenic diet and some of its variations may be an effective alternative treatment for drug-resistant epileptic patients with low side effects. Some even suggest that the diet should be used as a form of treatment alongside AED treatment when the epileptic condition is identified and diagnosed [28]. The goal of this review is to examine the ketogenic diet’s role in managing drug-resistant epilepsy and to highlight the impact of implementing the diet in the early stages of the disorder (Table 2).

## 3. Efficacy of the Ketogenic Diet on Drug-Resistant Epilepsy

The studies discussed in Table 2 present data that many drug-resistant patients achieve seizure reduction in 50–90% of epilepsy patients [12,20,29,30,32,34,42,43,44]. Some patients achieved up to a 100% reduction in seizures [33]. Infants, adolescents, and adults were evaluated in at least one study. Overall, infants were more responsive to ketogenic diets in promoting complete cessation of seizures than adults. This may be due to better compliance, as the ketogenic diet is a very restrictive diet, and caregivers attend to the food for infants. Patients with varying epileptic conditions and types of epilepsy were participants in the study. All studies demonstrated that the diet was effective in both types of epilepsy; however, there is still controversy within the field. The “controversy” stems largely from differences in methodology and among which patient populations responded best. Nei et al. [29] suggested that the diet may be more beneficial for generalized epileptic conditions in adults and adolescents. In a smaller study (n = 11) by Sirven et al. [32] from the same group, a strict medication stability throughout the trial period was maintained, whereas Nei et al. permitted the physicians to change the medication three months after starting the diet, a methodological difference that confounds the results about the efficacy of KD in seizure reduction reported by Nei et al. [29]. In contrast, Dressler et al. [42] reported greater effectiveness of KD in infants with focal epilepsy. Additionally, the variation in the ketogenic diet may need to be considered when prescribing a diet for epilepsy. The modified Atkins diet was found to be effective and suitable for adolescents and adults [42]. Seizure freedom was suggested to be more obtainable long-term for infants compared to any other age group by Dressler et al. [42], as supported by a review of 13 randomized controlled trials [29]. While lifelong side effects of the diet were not identified in these studies, Caraballo et al. found that seizures returned in some patients after the diet was discontinued [30].

All the studies reported positive outcomes on seizure control, and the presence of associated side effects was frequently observed. The majority of side effects were gastrointestinal-related issues, such as vomiting and constipation [12,29,32]. Each study had patients discontinue the diet shortly after initiation due to non-compliance from the patient and caregiver [12,32,33]. Some participants complained of low energy and hunger due to the restrictive diet [12,32]. In terms of the restrictiveness of the diet, there is an increased concern for malnutrition, especially in children. Most studies had resulted in weight loss in some patients; however, one study did not address any concern for weight loss or malnutrition [17,29,32]. Notably, Nei et al. [29] found that weight loss can be reversed by increasing total calories to meet metabolic demands for growth. All researchers agreed that malnutrition is an important side effect and recommended that it be monitored frequently, as it is a risk factor for poor growth in children [45]. Therefore, weight and other parameters, such as height, BMI, and regular laboratory panels (including lipids), would need to be frequently measured for patients on the ketogenic diet [12,29,32].

Studies have shown that interventions designed to improve motivation and adherence to nutrition-based therapies in children with epilepsy, such as the ketogenic diet and its variants, have had mixed but generally modest success [34,46,47]. Adherence remains a significant challenge, with less than half of children maintaining the ketogenic diet for one year and only about one-fifth for two years [48,49]. The most common reasons for discontinuation are unsatisfactory seizure control, patient or caregiver preference, and dietary intolerance, rather than adverse effects alone [12,43,50].

Parental stress is a notable barrier to adherence, particularly over the long term, and increases with the duration of dietary therapy. This underscores the need for ongoing psychosocial support for families to maximize adherence [44]. Less restrictive diets, such as the modified Atkins diet (MAD) and low glycemic index therapy (LGIT), are associated with better tolerability and comparable efficacy, and may improve adherence compared to the classic ketogenic diet KD [51].

The guidelines published by the French Association of Nutritionist Dietitians (AFDN) and the French Speaking Society of Clinical Nutrition and Metabolism (SFNCM) recommend that all adult patients admitted to hospital should undergo systematic nutritional risk screening at admission. For those identified at risk, a comprehensive nutritional assessment should be performed to guide individualized care plans. The guidelines emphasize the use of standard hospital diets for patients without specific nutritional needs and therapeutic diets tailored to clinical conditions (e.g., diabetes, renal failure, malnutrition) for those with specific requirements [52].

## 4. Anticonvulsant Mechanisms of Ketogenic Diet in Epilepsy

The metabolic bypass of impaired glucose transport and pyruvate dehydrogenase deficiency provides a clear therapeutic rationale for rare epileptic disorders [53]. Previous clinical and experimental studies on the broader efficacy of the KD in drug-resistant epilepsy reveal complex, multi-faceted anticonvulsant mechanisms [54]. Caloric restriction within the ketogenic diet redirects the metabolism to burn fats to generate ketone bodies, such as β-hydroxybutyrate (BHB) and acetoacetate (AAC), for energy production in neurons. This metabolic shift is proposed to stabilize the neuronal membranes, improve mitochondrial function, reduce oxidative stress, increase inhibitory (GABA) neurotransmitters, and activate potassium channels to hyperpolarize neurons [55].

The metabolic mechanism of the KD occurs through modulation of energy metabolism, where adenosine triphosphate (ATP) production shifts from glycolysis to fatty acid oxidation and ketone body utilization. Animal studies have shown that BHB improves mitochondrial respiration by stabilizing the ATP/O_2_ ratio through a reduction in reactive oxygen species generation [56]. Ketone bodies also serve as free radical scavengers and improve glutathione levels in the brain [56,57], thus reducing oxidative stress and decreasing inflammation. Neuroprotective mechanisms of the KD are considered by altering the composition of the gut microbiome and gut–brain axis, production of short-chain fatty acids, and production of neurotransmitters GABA and glutamate [58] by both the gut microbiome and the intestinal cells. BHB from KD promotes biosynthesis of neuro-inhibitory GABA from glutamate, a primary excitatory molecule in the brain [57,59]. Experimental evidence from *Drosophila* and mice suggests a role of BHB in the activation of ATP-dependent potassium channels (K_ATP_) [60,61]. The activation of K_ATP_ channels hyperpolarizes the neurons and reduces the electrical excitability to decrease seizures in DRE.

Despite the success in reducing seizures, the KD is associated with challenges. Limiting glucose over the long term alters three interconnected metabolic cycles: the folate cycle, the methionine cycle, and the trans-sulphuration pathway. The three pathways provide methyl groups for DNA/RNA synthesis, synthesis of vitamins (B2, B6, B12), and sulfur metabolites. A long-term KD may impact neuronal, renal and liver function and may lead to micronutrient deficiency [61,62], requiring individualized monitoring of diet [58].

## 5. Micronutrient Deficiencies in Epilepsy

Because the ketogenic diet is such a restrictive diet, micronutrient deficiencies are a common side effect and a major concern that needs to be corrected. In their study, Jortberg and Flemming (2014) evaluated the effects of the diet on micronutrient status and found that only five of the observed 28 essential micronutrients were adequately met [63]. This study monitored a food-based classical ketogenic diet at a 4:1 ratio. However, other studies indicate that less restrictive variations in the diet are associated with fewer concerns regarding micronutrient deficiencies, such as a 2:1 or 1:1 ratio [63,64]. Furthermore, patients on an enteral nutrition diet are also less at risk as the ketogenic formulas are fortified with the correct nutrients [63]. Pediatric and young adult patients are reportedly at the greatest risk for micronutrient concerns, as this population is still growing [8]. Poor growth and reduced bone health are often related to micronutrient deficiencies and can result in long-term consequences, including osteoporosis [8]. These side effects make monitoring micronutrient levels crucial.

Higher micronutrient risk is suggested to be related to a variety of factors. Low intakes of fruits, vegetables, complex carbohydrates, and dairy are often the cause of poor micronutrient status, as these food groups are abundant in a variety of vitamins and minerals [65]. Aside from this diet being incredibly restrictive, high parathyroid hormone activity and poor conversion of the active form of vitamin D are also observed in patients following these diets, which may be due to poor nutrient intake or drug–nutrient interactions from the patient’s regimen [65,66]. These changes can have detrimental effects on the micronutrients associated with bone health. When parathyroid hormone activity is high, osteoclasts are stimulated to extract calcium and phosphorus from the bone for release into the bloodstream [64,67]. Additionally, if vitamin D cannot be converted to its active form, osteoblasts are not stimulated to encourage the mineralization of bone from calcium and phosphorus [64,68,69]. Vitamin D also influences the synthesis of neurotransmitters like dopamine and serotonin by regulating key enzymes involved in their production [68]. Calcium and phosphorus play an important role inside the bone to help keep the bone strong, whereas vitamin D and magnesium help regulate calcium and phosphorus so that these minerals remain inside the bone [8]. Calcium is essential in neurotransmitters released at synaptic terminals, acting as a signal for vesicle fusion and communication between neurons. Magnesium modulates NMDA receptor activity and plays a critical role in maintaining synaptic plasticity, which is essential for learning and memory. Phosphorus, as a component of ATP, provides the energy needed for numerous enzymatic processes, including those involved in neurotransmitter synthesis and signal transduction. Therefore, vitamin D, calcium, phosphorus, and magnesium are important nutrients for bone health and require careful monitoring during the use of the ketogenic diet (Table 3) [8].

Aside from micronutrients for bone health, selenium and B vitamins were shown to be at risk for ketogenic patients. Selenium is a mineral with antioxidant properties that plays a role in protecting the heart muscle [77]. There are studies that show that selenium deficiency in ketogenic patients has resulted in heart problems and anemia [78]. Selenium levels appear to begin declining 6–12 months after initiating the diet, so close monitoring is necessary [79,80]. Regarding B vitamins, all B vitamins are a major concern for patients on the KD, recommended as MNT [81]. Vitamin B12 is among the least likely micronutrients to be deficient in epilepsy patients on the KD, with ideal levels in the mid to high range (500–900 pg/mL) [82]. However, it should still be monitored because a deficiency may cause neurological effects for those on vegan-based KD [83]. Vitamin B6 supplementation may be necessary depending on the epileptic condition [84,85]. Pyridoxine-dependent epilepsy requires high doses of vitamin B6 to reduce the occurrence of seizures from this condition [86]. For other conditions, vitamin B6 has also been studied to improve mood in epileptic patients; however, more research is required to better understand this phenomenon [84,86]. Folate supplementation is recommended to begin at the initiation of the diet, especially if a patient is taking anti-epileptic medications that can interact with folate absorption [82]. Monitoring of KD-induced folate and B12 deficiency is important for the development of megaloblastic anemias and other neurological symptoms. Despite contradictory reports [87], biotin and pantothenic acid deficiencies have been commonly observed in these patients, so supplementation may be warranted if deemed necessary through low serum levels [68,88]. Overall, virtually all the above-mentioned vitamins and minerals are at risk for deficiency in ketogenic patients, so close monitoring of serum lab values and supplementation as needed is an important consideration throughout the duration of this diet. Patients with epilepsy are also at increased risk of deficiencies due to AED-induced metabolic alterations and dietary restrictions [82]. In patients with epilepsy on the ketogenic diet, the prevalence of micronutrient inadequacies increased with age, confirming the negative effects of KD treatment on vitamins (A, B1, B2, B3, B6, B12, C, E, and folate) and minerals (calcium, iron, magnesium, phosphorus, and zinc) status despite supplementation [89]. This suggests the importance of personal monitoring of micronutrients and adjusting the supplementation dose accordingly during all steps of KD treatment [82,86]. Therefore, higher recommended intakes of these nutrients in patients with epilepsy can help maintain neural health, reduce the risk of deficiency-related complications (such as neuropathy and cognitive impairment), and may improve seizure control and quality of life. Regular monitoring and tailored supplementation are essential in this population [8,90].

Because ketogenic-friendly foods are limited, micronutrients are often recommended. A patient may be recommended to take a commercial multivitamin [8]. Multivitamins for ketogenic patients must be low in carbohydrates and should not negatively react with medications. It is important to note that these multivitamins have not been shown to meet all nutritional needs. For example, a study found that the use of a multivitamin did not help meet the magnesium needs [91]. Micronutrient status should be monitored through serum lab tests. If nutrient requirements are not adequately met through ketogenic-friendly foods or multivitamins, a high-potency supplement is prescribed to target and correct the identified deficiency [12,29].

## 6. Role of a Registered Dietitian

Much of the literature on the ketogenic diet indicates the complexity of medical nutrition therapy. Therefore, RD is necessary to safely and appropriately enforce the diet. An RD is an important member of the interdisciplinary team that reviews the overall health status of the patient to determine the best medical nutrition therapy by using evidence-based practice [92]. The RD will further personalize the diet according to the patient’s and caregiver’s needs. Since compliance was a common reason for diet discontinuation, the RD must provide proper education and diet plans to the patient and/or caregiver [39]. The addition of an RD in the interdisciplinary team is cost-effective [39]. The RD considers what medically fortified formulas and supplements are covered by insurance, if applicable [46], and will monitor and evaluate the patient in both inpatient and outpatient settings [93]. This would include frequent monitoring of the patient’s weight, compliance, and micronutrient status. The RD will adjust nutritional needs as necessary for the patient to reach optimal health while reducing seizure severity.

To optimize the implementation of the ketogenic diet, RDs must serve as strong advocates for their patients. An RD’s comprehensive assessment of a patient’s overall health status is used to develop an individualized dietary plan that aligns with clinical guidelines, while respecting the specific needs of the family. By adopting a patient-centered approach and meeting patients and families where they are, the clinicians are more likely to see sustained adherence to diet. Furthermore, an interdisciplinary strategy involving fully informed neurologists may promote a more comprehensive and effective treatment outcome in epilepsy patients.

## 7. Future Research

To better interpret the findings of this review, further examination of the factors contributing to seizure freedom is needed. While infants appear more likely to achieve seizure freedom [33,42], this success may be largely attributed to strict parental oversight and controlled administration; conversely, the reasons for the reduced response observed in older patients remain unclear. Future research should move beyond general efficacy to investigate specific barriers to success, such as the impact of gastrointestinal distress. For instance, given that manageable bowel movements require significant increases in fluid intake and fiber supplements, studies have analyzed how chronic constipation is a major but often underestimated side effect that influences long-term adherence to KD. More work is needed to determine whether dietary intolerance, rather than a lack of biological response, is what prevents older, more autonomous patients from achieving seizure freedom. Furthermore, an improved and updated list of the nutritional requirements for the epilepsy population is necessary. Current micronutrient recommendations for individuals following the ketogenic diet are based on dietary reference intakes for the general population [8], underscoring the need for future research to establish condition-specific guidelines.

## 8. Discussion

The synthesis of the current literature underscores that KD is not merely a metabolic alternative for rare glucose-processing disorders, but rather a potent neuromodulatory intervention capable of altering the excitatory-inhibitory balance of the brain. While the “metabolic bypass” remains a primary indication for specific pediatric populations, the broader efficacy seen in DRE across all age groups suggests that the diet’s success relies on a synergy of cellular hyperpolarization, neurotransmitter regulation, and reduced oxidative stress.

However, the clinical utility of the KD is frequently hampered by its restrictive nature, which presents a “therapeutic paradox”: the very metabolic shift required for seizure control simultaneously predisposes the patient to systemic nutritional risks. The findings of this literature review highlight that, while seizure reduction is often achieved, it frequently comes at the cost of altered folate and methionine cycles, potentially leading to long-term renal, hepatic, and bone health complications. This necessitates a shift in the clinical paradigm from viewing the KD as a “stand-alone” diet to viewing it as a complex pharmacological-grade intervention that requires rigorous biochemical monitoring.

Another criticism of KD therapy is a significant gap between clinical efficacy and long-term adherence. In adults, where compliance is notably lower than in pediatric cohorts, the transition to less restrictive variants like the modified Atkins diet (MAD) suggests that “maximal ketosis” may not always be the optimal target if it results in malnutrition or discontinuation. Therefore, the interpretation of clinical success must be expanded to include “nutritional stability” alongside “seizure freedom.”

Finally, the pivotal role of the registered dietitian (RD) emerges as the bridge between theory and practice. The RD’s involvement is not only supportive, but it is foundational to the diet’s safety profile. By proactively managing micronutrient supplementation and monitoring serum levels of Vitamin D, calcium, and selenium, the RD mitigates the inherent risks of the KD. Future research should focus on standardized supplementation protocols that can be integrated into the initial prescription of the KD to prevent deficiencies before they manifest clinically. In conclusion, the KD remains a cornerstone of epilepsy management, provided it is managed within an interdisciplinary framework that balances neuroprotection with comprehensive nutritional integrity.

## 9. Conclusions

This review is intended to promote the need for additional research in medical nutrition therapy, specifically for drug-resistant epilepsy. The overall literature review of diet trials suggests a positive outcome of ketogenic diets on seizure reduction for patients with epilepsy. Malnutrition may be a concern associated with the ketogenic diet, so regular monitoring of weight and serum micronutrient status is necessary, especially for pediatric and geriatric patients. Current research has demonstrated that patients on a ketogenic diet need greater than recommended supplementation of micronutrients; further research is needed to determine the exact dosages.

Despite its efficacy, the ketogenic diet remains underutilized in clinical practice. Healthcare providers prioritize advanced anti-seizure medications over the ketogenic diet, which is considered a last resort intervention or an alternative lifestyle. It is proven that a combination of medical nutrition therapy and drug/surgical treatment may result in the optimal impact on seizure occurrences. To increase use of the ketogenic diet, dietitians must advocate for patients by assessing overall health and guiding appropriate implementation. Collaboration between RDs and neurologists, supported by up-to-date knowledge and interdisciplinary care, can enhance epilepsy management with this manageable and effective therapy.

While this review aimed at presenting current research investigating the KD for epilepsy treatment, we do acknowledge some limitations to the review. The studies included varied study designs, patient populations, and some lacked assessment of bias. The KD diet has been found to help some patients find seizure freedom across multiple studies. However, further research is required to understand how these patients achieved seizure freedom.

## Figures and Tables

**Table 1:** Keywords used in PubMed search results for research published from the year 2000 onwards. Relevance is the presence of “epilepsy” or “epileptic” in the title of the paper.

Search Input	Number of Papers Screened	Number of Papers Relevance/Retained
Medical nutrition therapy AND epilepsy	1888	108/171
Ketogenic diet and epilepsy	2535	124/201
Clinical diets AND epilepsy	1671	115/176
Seizure AND medical nutritional therapy	997	96/155
Seizure AND clinical diets	1421	108/144

**Table 2:** Current clinical studies on the effects of the ketogenic diet in patients with drug-resistant epilepsy.

Study	Study Parameters	Success Rate	Limitations
Nei et al., 2014 [29]. n = 29	24-month evaluation of classic KD. adolescents and adults (n = 29). mean age 32.	52% of patients had seizure reduction. mean duration of 9 months.	Medications were allowed to be changed after 3 months of starting the diet [29]. Difficult to discern if the seizure reduction is due to diet or medication.
Caraballo et al., 2011 [30]. n = 216	Patients unresponsive to ≥2 anticonvulsants. diet as treatment. 1–20 years evaluation on classic KD. Most patients stayed on diet for 3 years.	22% of patients achieved seizure freedom. 56% of patients had at least a 75% reduction in seizures. 93 patients out of 216 were able to significantly reduce medication use.	10 patients who reached seizure freedom and many patients who reached seizure reduction were given topiramate [30]. The medication is a powerful anti-epileptic drug [31], so it could have altered the results.
Sirven et al., 1999 [32]. n = 11	11 adults with drug-resistant epilepsy on classic KD.	After 8 months on diet, 6 patients had at least a 50% reduction in seizures. 4 discontinued the study.	Nonrandomized and uncontrolled study. Neal et al. [33] implemented this study design for the same research question.
Neal et al., 2008 [33]. n = 145	145 children aged 2–16 years old with drug-resistant epilepsy on classic KD. randomized controlled trial.	38% of patients had ≥50% reduction in seizures. 7% of patients had more than 90% reduction in seizures.	Lack of blinding may lead to potential bias. A systematic review [34] included this study and rated its risk for bias low.
El-Shafie et al., 2023 [12].	40 children aged 4–144 months old with drug-resistant epilepsy put on D or modified Atkins Diet (MAD). 30 participants completed the trial.	60% of patients in KD and 46.7% in MAD group experienced seizures. Other 40% and 53.3% have ≥50% reduction in seizures.	Small number of participants. Excluded from meta-analysis by Yakemow et al., 2026 [13]. Reason for exclusion is not clear. Potentially due to use of two types of diet.
Kverneland et al., 2023 [35]. n = 39	MAD (n = 19) diet or control diet (n = 20) for 12 weeks. Self-reported HRQOL evaluated based on QOLIE-89.	QOLIE-89 increased by 10 points in MAD group compared to controls (*p* = 0.002). No relationship with 50% reduction in seizure.	Low number of subjects and short duration of the trial.
Anand et al., 2025 [36]. n = 91	modified Atkins diet (MAD) and low glycemic index treatment (LGIT).	MAD is superior in seizure reduction, 60.7 ± 41.3% vs. 57 ± 39.4% in LGIT group.	20% of subjects dropped out. One person evaluated surveys and seizure reductions.
Manral et al., (2023) [37]. n = 160	Randomized to receive SDT plus MAD (intervention arm, n = 80) or SDT alone (control arm, n = 80) for 6 months. Primary outcome ≥ 50% reduction in seizure frequency.	≥50% reduction in seizure observed in 26.2% of intervention group vs. 2.5% of control groups.	Non-blinded. 31% attrition rate in MAD + SDT group.
IJff et al., (2016) [38] n = 50	Classic KD (n = 28) and care-as-usual (n = 22) as control for 4 months duration.	Improved behavior and cognition in KD group.	Both classic KD and MCT KD diets were used. Use of proxy measures to assess impact of KD.
Kossoff et al., (2008) [39]. n = 30	Adults (n = 30) patients on MAD for 6 months.	47% of patients showed 50% seizure reduction after 1–3 months on MAD. 33% showed after 6 months.	Open label. No controls
McDonald et al., 2018 [40]. n = 80	MAD alone vs. MAD supplemented with classic KD for 6 months.	Over 50% subjects showed ≥ 50% reduction in seizure. Early supplementation of MAD with ketogenic formula increases compliance.	Open-label, non-blinded. A larger study is required.
Neal et al., (2009) [41]. n = 61	61 children on classical KD (n = 45) and MCT (49) diet.	No significant differences in seizure reductions in both groups.	Lack of double blinding. Use of self-reported total number of seizures.
Dressler et al., 2015 [42]. n = 127	127 children aged 2.86 years ± 3.1 (min. 0.0–max. 16.8) with epilepsy on KD. Evaluated for efficacy and long-term use.	Early introduction of diet soon after diagnosis provides higher chance of reaching seizure freedom. 61% of patients resulted in seizure reductions. 27% seizure freedom.	Cross-sectional study that would have benefited from a randomized controlled trial. Martin-McGill et al. [34] suggested that some trials of ketogenic diet interventions may have gained more efficacy if they used a randomized controlled trial study design.

HRQOL: health-related quality of life, QOLIE-89: quality of life in epilepsy inventory-89, MAD: modified Atkins diet, KD: ketogenic diet, MCT: medium-chain triglyceride.

**Table 3:** Key micronutrients important for bone and neural health.

Micronutrient	Effect of Poor Nutrient Status	Suggested Recommendation
Vitamin D	Bone fractures, rickets in pediatric patients, and osteoporosis in adults [32]. Patients with epilepsy are at high risk for vitamin D deficiency due to chronic use of enzyme-inducing AEDs and lifestyle factors like limited sun exposure. prevalence rates exceeding 40% in some populations [70,71].	Supplementation of 400 IU over the DRI, monitor levels frequently [64].
Calcium	Demineralization of bone and hyperstimulation of nervous system leading to worsening seizures [32]. Patients with epilepsy, especially those with generalized forms, often have lower serum calcium levels compared to healthy controls [22].	Prescription supplement that includes calcium with vitamin D3: 1000–1200 mg/day [64].
Phosphorus	Demineralization of bone [72]. Chronic phosphorus deficiency in epilepsy patients, especially those with severe disability, malnutrition, or on certain AEDs. Lead to life-threatening complications like neuromuscular and cardiovascular dysfunction and may worsen neurological status. AEDs, such as valproate and phenytoin, are linked to hypophosphatemia, increasing the risk of osteopenia and fractures [73].	Supplementation should not be taken together with calcium. Recommendation: 700 mg/day [64].
Magnesium	Parathyroid hormone imbalance. Poor regulation of calcium and phosphorus, and deficiency may worsen seizures [36]. Magnesium deficiency increases neuronal excitability and lowers the seizure threshold, potentially triggering seizures or status epilepticus; it should be considered in refractory epilepsy, especially with other electrolyte imbalances [74].	Prescription supplement: 310–420 mg/day [36].
Vitamin B	Vitamin B deficiencies can impair neurotransmitter synthesis (e.g., GABA) and brain function. Increased the risk of seizures and neurological complications. Can impair DNA synthesis and myelin formation. Deficiencies or inborn errors in B vitamin metabolism can directly cause or exacerbate epilepsy (e.g., pyridoxine-dependent epilepsy)	Supplementation with B vitamins (especially folate and B12) has been shown to reduce hyperhomocysteinemia and improve mood. May reduce seizure frequency in some cases.
Zinc	Modulates synaptic activity and GABA function. Lower serum zinc levels are observed in patients with drug-resistant or intractable epilepsy compared to well-controlled epilepsy and healthy controls. Essential for neuronal excitability and synaptic function; zinc deficiency may increase seizure susceptibility. Both deficiency and excess can have pro- or anticonvulsant effects depending on context and concentration [75].	Supplement with zinc-rich food. Standard adult supplementation regimen of 20–40 mg elemental zinc daily for epilepsy patients with deficiency [76].

## Data Availability

No new data were created or analyzed in this study.
